# Heparin-based versus bivalirudin-based anticoagulation in pediatric extracorporeal membrane oxygenation: A systematic review

**DOI:** 10.3389/fmed.2023.1137134

**Published:** 2023-03-14

**Authors:** Carlos A. Valdes, Omar M. Sharaf, Mark S. Bleiweis, Jeffrey P. Jacobs, Mohammed Mumtaz, Ramy M. Sharaf, Eric I. Jeng, Giles J. Peek

**Affiliations:** Department of Surgery and Pediatrics, University of Florida, Gainesville, FL, United States

**Keywords:** anticoagulation, bivalirudin, extracorporeal membrane oxygenation (ECMO), pediatric, direct thrombin inhibitor

## Abstract

**Introduction:**

Optimal anticoagulation therapy is essential for the prevention of thrombotic and hemorrhagic complications in pediatric patients supported with extracorporeal membrane oxygenation (ECMO). Recent data have demonstrated bivalirudin has the potential to surpass and replace heparin as the anticoagulant of choice.

**Methods:**

We conducted a systematic review comparing the outcomes of heparin-based versus bivalirudin-based anticoagulation in pediatric patients supported on ECMO to identify the preferred anticoagulant to minimize bleeding events, thrombotic complications, and associated mortality. We referenced the PubMed, Cochrane Library, and Embase databases. These databases were searched from inception through October 2022. Our initial search identified 422 studies. All records were screened by two independent reviewers using the Covidence software for adherence to our inclusion criteria, and seven retrospective cohort studies were identified as appropriate for inclusion.

**Results:**

In total, 196 pediatric patients were anticoagulated with heparin and 117 were anticoagulated with bivalirudin while on ECMO. Across the included studies, it was found that for patients treated with bivalirudin, trends were noted toward lower rates of bleeding, transfusion requirements, and thrombosis with no difference in mortality. Overall costs associated with bivalirudin therapy were lower. Time to therapeutic anticoagulation varied between studies though institutions had different anticoagulation targets.

**Conclusion:**

Bivalirudin may be a safe, cost-effective alternative to heparin in achieving anticoagulation in pediatric ECMO patients. Prospective multicenter studies and randomized control trials with standard anticoagulation targets are needed to accurately compare outcomes associated with heparin versus bivalirudin in pediatric ECMO patients.

## Introduction

In this review, we will briefly highlight the issues unique to pediatric versus adult extracorporeal membrane oxygenation (ECMO) and summarize the mechanism of action of heparin and bivalirudin before identifying and summarizing the literature comparing the outcomes of heparin-based versus bivalirudin-based anticoagulation in pediatric patients (≤18 years) supported on ECMO.

There are important physiological considerations that differentiate the management of pediatric ECMO patients from adult ECMO patients. Specifically, the field of developmental hemostasis may explain differences in physiological response to anticoagulant medications used during ECMO support between pediatric and adult patients. Developmental hemostasis refers to a concept that was coined by Andrew and colleagues in the 1980s—their group demonstrated age-related changes in the levels of various coagulation proteins (1, 2). While early studies suggested that neonates reached adult levels of various coagulant proteins by six months of age, a later study by their group confirmed differences in coagulant protein levels between pediatric and adult patients, including lower levels of prothrombin, factor V, factor VII, factor IX, factor X, factor XI, and factor XII in pediatric patients (3). Others have supported these findings, showing lower prothrombin availability and a lower conversion rate of prothrombin to thrombin in pediatric patients (4). These age-related differences in hemostasis are additionally reflected by the differences in pediatric and adult reference ranges for antithrombin (AT) and activated partial thromboplastin time (aPTT) lab values, which must be considered when monitoring anticoagulation. Yet, while it is understood that hematological age-related differences in coagulation exist, there remains uncertainty about the extent of these changes and about how these differences may impact anticoagulant activity in pediatric patients requiring ECMO support.

Several *in vivo* and *in vitro* studies have shown age-dependent physiological differences in response to anticoagulant medications secondary to these age-related changes in hemostasis (5, 6). Heparin has historically been used for anticoagulation in ECMO patients, yet age-related differences account for differences in its therapeutic effect in pediatric versus adult patients. The mechanism of action of heparin is largely driven by its interaction with AT. AT, a naturally occurring anticoagulant protein, counteracts the coagulation cascade to maintain hemostasis and limit thrombosis. AT acts primarily by inhibiting thrombin and factor Xa (7). Heparin functions primarily by binding to AT and increases its activity to minimize coagulation (7), yet this complex only inhibits free thrombin (8). Though less significant, heparin administration also increases endothelial production of tissue factor pathway inhibitor (TFPI) and heparin itself increases the activity of this anticoagulant protein by facilitating its binding to and inhibition of factor Xa (9, 10). Differences in the levels of these procoagulant and anticoagulant proteins between pediatric and adult patients incompletely explain the age-dependent differences in heparin’s therapeutic effect. While pediatric patients have lower thrombin levels, which would suggest that less inhibition is needed to achieve a similar therapeutic effect, these patients often require increased doses of heparin compared to adults. This is partially explained by the lower serum levels of AT observed in pediatric patients (11); however, these differences limit the predictability of heparin’s therapeutic effect. Additionally, patients can become resistant to its anticoagulative effects due to the diminishing amount of AT that heparin binds to and uses for its continued function (12), which is especially pronounced in children given their lower levels of AT. In addition to these limitations, heparin-induced thrombocytopenia (HIT) is a complication that can also occur when platelets are activated in response to heparin, paradoxically causing thrombosis (13).

As many as 3.6% of ECMO patients develop HIT, which is associated with a mortality rate of up to 60% in these patients (14). The prevalence of HIT is reduced in pediatric patients (15), though it is still associated with significant complications. There are two mechanisms by which HIT has been described to occur—type 1 and type 2. In both, heparin administration activates platelets via different mechanisms and with different consequences. Specifically, in type 1 HIT, which is the more frequent mechanism but with less significant consequences (16, 17), heparin directly induces platelet aggregation but is not associated with increased risk of thrombosis (18). Thus, it is also known as non-immune heparin-associated thrombocytopenia. Type I HIT is less severe with mild thrombocytopenia occurring within two days of heparin initiation—this is self-limited and resolves without cessation of heparin (17). Whereas in type 2 HIT, an immune response occurs against platelet factor-4 (PF4)/heparin complexes which form naturally as endogenous PF4 binds and neutralizes heparin (19). Immunoglobulin G (IgG) binding to these PF4/heparin complexes forms an immunocomplex which binds to and activates platelets, causing thrombosis. Type II HIT is more severe with significant thrombocytopenia five to ten days after heparin initiation and requires immediate cessation of heparin (20). Given the clinical impact of this complication, this is an important limitation of heparin that must be considered when choosing anticoagulation in pediatric ECMO patients.

Due to the limitations of heparin, direct thrombin inhibitors such as bivalirudin are increasingly being used for anticoagulation in pediatric patients (21). Bivalirudin’s mechanism of action involves direct inhibition of thrombin activity, without the need for AT, making it a more predictable option for consistent anticoagulation—especially in pediatric patients who have varying levels of AT and thrombin. Additionally, its therapeutic effect is similar between adult and pediatric patients, though the effect of a loading dose is variable and incompletely understood (22). Another advantage over heparin includes bivalirudin’s ability to bind to and inhibit both free and clot-bound thrombin. For these reasons, bivalirudin is an attractive alternative to heparin in pediatric patients requiring ECMO though it has limitations as well. Elimination of bivalirudin is achieved via proteolytic cleavage by thrombin and renal excretion (23); however, in contrast to heparin, bivalirudin has no specific reversal agent although the half-life of bivalirudin is significantly shorter than that of heparin. Despite the benefits of bivalirudin compared to heparin, its use is presently limited due to the lack of prospective randomized studies supporting its use.

In addition to the development of the hemostatic system as they grow, babies and children requiring ECMO can vary greatly in size. Patients under the age of 18 years and from around 2 kg to in excess of 150 kg may all be in need of ECMO. Clearly, these patients will need to have their ECMO cannulas and circuits tailored to their size and specific oxygen consumption, which also reduces with age (24). ECMO circuits may vary from 1/4” tubing with a roller pump, through 1/2” tubing with a centrifugal pump to adult integrated pump-oxygenator devices with 3/8” tubing stepped down to 1/4” with a bridge to maintain adequate flow in the oversized oxygenator (25, 26). Oxygenators are also sized according to the expected blood flow from 800 cm^2^ up to around 7,000 cm^2^. The heterogeneity inherent in caring for these patients increases both the difficulty of ensuring adequate anticoagulation and evaluating the published evidence.

## Methods

In accordance with the Preferred Reporting Items for Systematic Reviews and Meta-Analyses (PRISMA), we conducted a systematic review of the literature comparing outcomes of heparin-based anticoagulation versus bivalirudin-based anticoagulation in pediatric patients (≤18 years) supported on ECMO. Databases referenced include PubMed, Cochrane Library, and Embase. These databases were searched from inception through 29 October 2022. The search terms were as follows: (bivalirudin OR bivalitroban OR angiomax OR angiox OR hirulog) AND (heparin OR unfractionated heparin OR UFH) AND (extracorporeal membrane oxygenation OR extracorporeal OR membrane oxygenation OR ECMO OR extracorporeal cardiopulmonary resuscitation OR ECPR). All records were imported into the Covidence systematic review software for screening. Two reviewers (CAV and OMS) independently screened titles and abstracts, and then screened full texts that adhered to the inclusion criteria.

Full-text articles that were available in English and that offered direct comparisons of primary heparin-based anticoagulation versus primary bivalirudin-based anticoagulation in pediatric patients (≤18 years) supported with ECMO were included. Specifically, inclusion criteria were as follows: (1) pediatric patients ≤18 years old, (2) comparison of management with heparin-based anticoagulation versus bivalirudin-based anticoagulation, and (3) patients supported on ECMO. We excluded non-human studies and case reports/series.

## Results

### Search outcome

**FIGURE 1:**
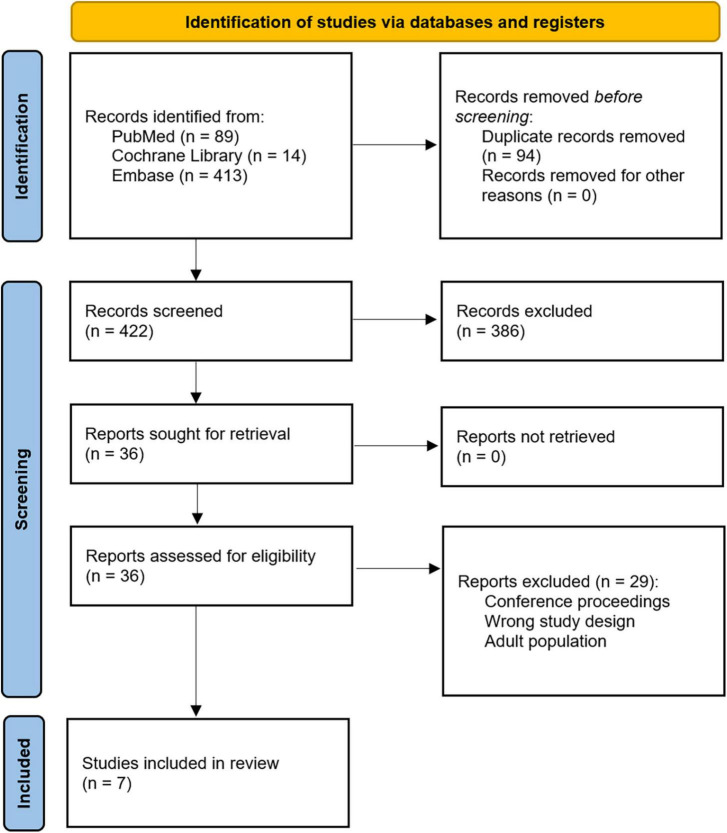
Preferred reporting items for systematic reviews and meta-analyses (PRISMA) flow diagram.

Our initial search query from the referenced databases returned 516 studies. After removal of duplicates, 422 records underwent title and abstract screening by two independent reviewers (CAV and OMS) using the Covidence software. Conflicts were discussed and resolved after mutual agreement. Title and abstract screening identified 36 records for full-text review. After full-text review, seven studies were selected for inclusion (Figure 1). The references for each of these seven records were also independently reviewed, and no additional articles were eligible for inclusion. All seven articles were retrospective cohort studies. Relevant variables that were consistently reported across articles were chosen, and data were extracted. These studies are summarized in Table 1. Variables of interest were stratified as follows: (1) Baseline characteristics (age, sex, and weight), (2) ECMO details (ECMO duration, type of support, indication for support, and cannulation strategy), (3) Time to therapeutic anticoagulation, (4) Transfusion requirements and bleeding events, (5) Thrombotic complications, (6) Mortality, and (7) Anticoagulation and ECMO costs.

**TABLE 1:** Overview of studies and variables recorded.

References	Study type	Sample size		Referenced variables						
		**Bival**	**UFH**	**Baseline characteristics^a^**	**ECMO details^b^**	**Time to therapeutic anticoagulation**	**Transfusion requirements and bleeding^c^**	**Thrombotic complications**	**Mortality**	**Costs**
Ranucci et al. (27)^d^	Retrospective cohort	5	5	Yes	Yes	No	No	Yes	Yes	Yes
Hamzah et al. (28)	Retrospective cohort	16	16	Yes	Yes	Yes	Yes	Yes	Yes	Yes
Schill et al. (32)	Retrospective cohort	14^e^	34^e^	Yes	Yes	No	Yes	Yes	Yes	No
Machado et al. (29)	Retrospective cohort	18	14	Yes	Yes	Yes	Yes	Yes	Yes	No
Kaushik et al. (31)	Retrospective cohort	8	27	Yes	Yes	Yes	Yes	No	Yes	No
Seelhammer et al. (30)	Retrospective cohort	24	65	Yes	Yes	No	Yes	Yes	Yes	No
Rabinowitz et al. (33)	Retrospective cohort	32	35	Yes	Yes	No	No	No	Yes	No

Bival, bivalirudin; ECMO, extracorporeal membrane oxygenation; UFH, unfractionated heparin.

^a^Baseline characteristics include patient age, sex, and weight. Studies may not have included all of these baseline characteristics.

^b^ECMO details include ECMO duration, type of support, indication for support, and cannulation strategy. Studies may not have included all of these ECMO details variables.

^c^Transfusions and bleeding includes comparisons of blood product utilization and of bleeding events. Studies may not have included all of these variables.

^d^Study provides data broken down by the anticoagulant used but only provides statistical comparisons including adult and pediatric patients together. Data in this table and throughout this review summarize the pediatric data without including statistical comparisons.

^e^Represents number of ECMO runs, rather than number of patients. This study had a total of 56 ECMO runs in 54 pediatric patients. There was a third group of patients who were switched from UFH to bivalirudin (*n* = 8 ECMO runs), not included here.

### Baseline characteristics

All seven studies included in this review discussed baseline characteristics, including age, sex, and weight (Table 2) (27–33). Age was reported in six studies (28–33), sex was reported in five studies (28–31, 33), and weight was reported in six studies (27–29, 31–33). There were no significant differences in age, sex, or weight between pediatric ECMO patients anticoagulated with heparin versus bivalirudin across all studies reporting these metrics. Patients were young with most studies reporting mean or median age of less than 18 months. Sex was well distributed across studies with a relatively equal distribution of male and female patients. Lastly, patients were small with most studies reporting a mean or median weight of less than 15 kg.

**TABLE 2:** Baseline patient characteristics.

Study	Sample size	Heparin	Bivalirudin	*p*-Value
**Age** ^a,b^				
Seelhammer et al. (30)	Heparin: *n* = 65 Bivalirudin: *n* = 24	<1 year: 42 (65)	<1 year: 11 (46)	0.21
		1–4 years: 12 (18)	1–4 years: 8 (33)	
		5–17 years: 11 (17)	5–17 years: 5 (21)	
Hamzah et al. (28)	Heparin: *n* = 16 Bivalirudin: *n* = 16	59 (0–212)	31 (0–99)	0.41
Machado et al. (29)	Heparin: *n* = 14 Bivalirudin: *n* = 18	39.8 ± 76.1	36.0 ± 58.8	0.36
Kaushik et al. (31)	Heparin: *n* = 27 Bivalirudin: *n* = 8	4.0 (0.5, 92.0)	0.6 (0.0, 80.0)	N/A
Schill et al. (32)	Heparin: *n* = 34 Bivalirudin: *n* = 14	16.3 (4.8, 143.7)	5.5 (3.7, 79.6)	0.23^c^
Rabinowitz et al. (33)	Heparin: *n* = 35 Bivalirudin: *n* = 32	13.46 (4.54, 118.48)	4.08 (2.34, 16.63)	0.21
**Sex (female)**				
Seelhammer et al. (30)	Heparin: *n* = 65 Bivalirudin: *n* = 24	30 (46)	11 (46)	0.98
Hamzah et al. (28)	Heparin: *n* = 16 Bivalirudin: *n* = 16	8 (50)	10 (62.5)	0.47
Machado et al. (29)	Heparin: *n* = 14 Bivalirudin: *n* = 18	5 (36)	11 (61)	0.15
Kaushik et al. (31)	Heparin: *n* = 27 Bivalirudin: *n* = 8	12 (44.4)	5 (62.5)	N/A
Rabinowitz et al. (33)	Heparin: *n* = 35 Bivalirudin: *n* = 32	17 (48.6)	13 (40.6)	0.52
**Weight (kg)** ^b^				
Hamzah et al. (28)	Heparin: *n* = 16 Bivalirudin: *n* = 16	15.4 (2.7–71)	8.5 (2.8-43)	0.32
Machado et al. (29)	Heparin: *n* = 14 Bivalirudin: *n* = 18	14.9 ± 22.2	19.9 ± 35.5	0.73
Schill et al. (32)	Heparin: *n* = 34 Bivalirudin: *n* = 14	10 (5, 42)	6 (5, 20)	0.26^c^
Kaushik et al. (31)	Heparin: *n* = 27 Bivalirudin: *n* = 8	6.2 (3.9, 26.4)	4.0 (2.6, 16.9)	N/A
Rabinowitz et al. (33)	Heparin: *n* = 35 Bivalirudin: *n* = 32	10.65 (5.20, 36.58)	5.64 (4.20, 11.50)	0.15
Ranucci et al. (27)	Heparin: *n* = 5 Bivalirudin: *n* = 5	3 (2.7-15)	7 (2.6-45)	N/A

^a^Displayed in months unless otherwise specified.

^b^Data displayed as mean ± standard deviation, median (interquartile range, i.e., 25th percentile, 75th percentile), or median (range, i.e., minimum–maximum).

^c^Schill et al. compares three groups: heparin, bivalirudin, and switched. The *p*-value represents a comparison between all three groups rather than between heparin versus bivalirudin.

### ECMO details

Details regarding ECMO support, including ECMO type, indication, cannulation strategy, and/or duration, were reported in all seven studies (Table 3) (27–33). All studies reported ECMO type with no differences in ECMO type across all studies—most patients were supported with veno-arterial (V-A) or veno-venous (V-V) ECMO, and few patients were supported with V-A-V or V-V-A ECMO. Five studies reported indications for ECMO and found no differences in indications for support between heparin and bivalirudin cohorts (27, 30–33). The most common ECMO indications reported included post-cardiotomy, respiratory failure, and extracorporeal cardiopulmonary resuscitation. Out of four studies reporting cannulation strategy, only two reported a statistical comparison. The larger of these studies—a single-institutional analysis of 65 heparin patients and 24 bivalirudin patients—found a higher rate of central cannulation in the heparin cohort (82% [*n* = 53] vs. 58% [*n* = 14], *p* = 0.02) (30). Meanwhile, Machado and colleagues found no difference in the cannulation strategy used (central versus peripheral) among their cohort of 32 patients (14 heparin, 18 bivalirudin) (29).

**TABLE 3:** ECMO details.

Study	Sample size	Heparin	Bivalirudin	*p*-Value
**ECMO, type**				
Seelhammer et al. (30)	Heparin: *n* = 65 Bivalirudin: *n* = 24	VA: 61 (94)	VA: 20 (83)	0.12
		VV: 4 (6)	VV: 4 (17)	
Hamzah et al. (28)	Heparin: *n* = 16 Bivalirudin: *n* = 16	VA: 16 (100)	VA: 13 (81.2)	0.23
		VV: 0 (0)	VV: 3 (18.8)	
Machado et al. (29)	Heparin: *n* = 14 Bivalirudin: *n* = 18	VA: 12 (86)	VA: 18 (100)	0.25
		VV: 1 (7)	VV: 0 (0)	
		Hybrid: 1 (7)	Hybrid: 0 (0)	
Schill et al. (32)	Heparin: *n* = 34 Bivalirudin: *n* = 14	VA: 23 (68)	VA: 9 (64)	1^c^
		VV: 11 (32)	VV: 5 (36)	
Kaushik et al. (31)	Heparin: *n* = 27 Bivalirudin: *n* = 8	VA: 24 (88.9)	VA: 6 (75)	N/A
		VV: 3 (11.1)	VV: 1 (12.5)	
		VAV: 0 (0)	VAV: 1 (12.5)	
Rabinowitz et al. (33)	Heparin: *n* = 35 Bivalirudin: *n* = 32	VA: 21 (60)	VA: 22 (69)	0.56
		VV: 13 (37)	VV: 8 (25)	
		VAV: 0 (0)	VAV: 1 (3)	
		VVA: 1 (3)	VVA: 1 (3)	
Ranucci et al. (27)	Heparin: *n* = 5 Bivalirudin: *n* = 5	VA: 5	VA: 5	N/A
**ECMO, indications**				
Seelhammer et al. (30)	Heparin: *n* = 65 Bivalirudin: *n* = 24	Post cardiotomy: 18 (28)	Post cardiotomy: 3 (13)	0.27
		Cardiac: 18 (28)	Cardiac: 6 (25)	
		Respiratory: 13 (20)	Respiratory: 9 (38)	
		ECPR: 16 (25)	ECPR: 6 (25)	
Schill et al. (32)	Heparin: *n* = 34 Bivalirudin: *n* = 14	Post cardiotomy shock: 9 (26)	Post cardiotomy shock: 7 (50)	0.57^c^
		Respiratory failure: 12 (35)	Respiratory failure: 5 (36)	
		Cardiogenic shock, unrepaired CHD: 5 (15)	Cardiogenic shock, unrepaired CHD: 0 (0)	
		Cardiogenic shock, other cause: 8 (24)	Cardiogenic shock, other cause: 2 (14)	
Kaushik et al. (31)	Heparin: *n* = 27 Bivalirudin: *n* = 8	Respiratory indication: 5 (18.5)	Respiratory indication: 6 (75.0)	N/A
		Cardiac indication: 18 (66.7)	Cardiac indication: 1 (12.5)	
		ECPR: 4 (14.8)	ECPR: 1 (12.5)	
Rabinowitz et al. (33)	Heparin: *n* = 35 Bivalirudin: *n* = 32	Acute respiratory distress syndrome/lower respiratory tract infection: 13 (37)	Acute respiratory distress syndrome/lower respiratory tract infection: 12 (38)	N/A
		Intrinsic lung disease/asthma: 3 (8)	Intrinsic lung disease/asthma: 2 (6)	
		Intoxication: 3 (8)	Intoxication: 0 (0)	
		Cardiac arrest: 2 (6)	Cardiac arrest: 2 (6)	
		Post-pericardiotomy: 9 (26)	Post-pericardiotomy: 10 (31)	
		Unrepaired CHD: 3 (8)	Unrepaired CHD: 1 (3)	
		Acquired heart disease: 1 (3)	Acquired heart disease: 4 (13)	
		Transplant rejection: 1 (3)	Transplant rejection: 1 (3)	
Ranucci et al. (27)	Heparin: *n* = 5 Bivalirudin: *n* = 5	Post cardiotomy: 5 (100)	Post cardiotomy: 5 (100)	N/A
**Cannulation strategy: central vs. peripheral**				
Seelhammer et al. (30)	Heparin: *n* = 65 Bivalirudin: *n* = 24	Central: 53 (82)	Central: 14 (58)	0.02
		Peripheral: 12 (18)	Peripheral: 10 (42)	
Hamzah et al. (28)	Heparin: *n* = 16 Bivalirudin: *n* = 16	Central: 3 (19)	Central: 4 (25)	N/A
		Peripheral: 13 (81)	Peripheral: 12 (75)	
Machado et al. (29)	Heparin: *n* = 14 Bivalirudin: *n* = 18	Central: 7 (50)	Central: 11 (61)	0.53
		Peripheral: 7 (50)	Peripheral: 7 (39)	
Ranucci et al. (27)	Heparin: *n* = 5 Bivalirudin: *n* = 5	Central: 5 (100)	Central: 5 (100)	N/A
		Peripheral: 0 (0)	Peripheral: 0 (0)	
**ECMO duration** ^a,b^				
Seelhammer et al. (30)	Heparin: *n* = 65 Bivalirudin: *n* = 24	7.2 (4.2, 18.6) days	5.3 (2.7, 13.8) days	0.19
Kaushik et al. (31)	Heparin: *n* = 27 Bivalrudin: *n* = 8	6.0 (3.0, 9.0) days	12.0 (5.5, 23.5) days	0.144
Hamzah et al. (28)	Heparin: *n* = 16 Bivalirudin: *n* = 16	114 (32–419)	98.8 (46–363)	0.95
Machado et al. (29)	Heparin: *n* = 14 Bivalirudin: *n* = 18	154.8 ± 107.7	166.6 ± 65.1	0.60
Schill et al. (32)	Heparin: *n* = 34 Bivalirudin: *n* = 14	3.3 (2.1, 6.2) days	11.0 (6.2, 23.1) days	0.001^c^
Rabinowitz et al. (33)	Heparin: *n* = 35 Bivalirudin: *n* = 32	152.47 (90.09, 310.17)	227.60 (136.68, 418.65)	0.83
Ranucci et al. (27)	Heparin: *n* = 5 Bivalirudin: *n* = 5	90 (16–124)	110 (87–234)	N/A

Values are presented as *n* (%) unless otherwise specified.

^a^Displayed in hours unless otherwise specified.

^b^Data displayed as mean ± standard deviation, median (interquartile range, i.e., 25th percentile, 75th percentile), or median (range, i.e., minimum–maximum).

^c^Schill et al. compares three groups: heparin, bivalirudin, and switched. The *p*-value represents a comparison between all three groups rather than between heparin versus bivalirudin.

Extracorporeal membrane oxygenation duration was reported in all seven studies. Only one of these studies reported a significant difference in ECMO duration between the cohorts evaluated (32). This study was a single-center analysis by Schill et al. (32) that included a third group of patients in their analysis of ECMO duration: patients who were switched from heparin to bivalirudin. Among 34 ECMO runs in heparin patients, 14 ECMO runs in bivalirudin patients, and 8 ECMO runs in patients who were switched from heparin to bivalirudin, median (interquartile range) duration of ECMO support was significantly different between groups (*p* = 0.001). Duration of support was 3.3 (2.1, 6.2) days in the heparin cohort, 11.0 (6.2, 23.1) days in the bivalirudin cohort, and 10.3 (8.3, 18.3) days in the cohort of patient who switched anticoagulant. In this review, the longest reported median duration of pediatric ECMO support was 7.2 (4.2, 18.6) days (30) across heparin cohorts and 12.0 (5.5, 23.5) days (31) across bivalirudin cohorts.

### Time to therapeutic anticoagulation

In this review, three of seven studies (Table 4) directly compared time to reach therapeutic anticoagulation between pediatric patients receiving heparin or bivalirudin during ECMO (28, 29, 31). These studies differed in their findings, but also had different therapeutic anticoagulation targets. In the study by Kaushik et al. (31) comparing 8 patients anticoagulated with bivalirudin and 27 patients anticoagulated with heparin at a single institution, there was no difference in time to reach therapeutic anticoagulation (median [IQR]: 14.5 [6.7, 16] vs. 12 [5.75, 26], respectively; *p* = 0.37). Anticoagulation targets in their study included an activated clotting time (ACT) of 200–240 s and/or a goal aPTT of 60–90 s for the heparin group and a target aPTT of 60–90 s for the bivalirudin group. While Machado and colleagues’ study also found no significant difference in time to reach therapeutic anticoagulation, the heparin group trended toward a shorter time and almost reached significance (mean ± SD: 12.54 ± 9.96 vs. 21.06 ± 12.53 h, *p* = 0.06) (29). This was a similarly sized single-center study with a well-balanced distribution of patients (18 patients received bivalirudin; 14 patients received heparin). However, in this study, the anticoagulation target goals were left to the discretion of each individual intensivist and surgeon. In contrast to both studies, the analysis by Hamzah and colleagues (28), which was also well-balanced and similarly sized (16 patients received bivalirudin; 16 patients received heparin), found that patients anticoagulated with bivalirudin had a significantly shorter time to reach therapeutic anticoagulation (median [range]: 11 [4–56] vs. 29 [5–128] h, *p* = 0.01). Again, the therapeutic anticoagulation goals differed, with a bivalirudin aPTT goal of 58–78 s and a heparin goal of (1) anti-Xa assay measurements of 0.3–0.7 international units/ml, (2) aPTT of 60–80 s, and/or (3) ACT of 180–200 s. These findings suggest inter-site variation in anticoagulation goals and time to reach therapeutic anticoagulation and suggest that neither anticoagulant is clearly superior in reaching therapeutic anticoagulation more quickly.

**TABLE 4:** Time to reach therapeutic anti-coagulation.

Study	Sample size	Heparin	Bivalirudin	*p*-Value
**Time to reach therapeutic anti-coagulation** ^a^ **, h**				
Hamzah et al. (28)	Heparin: *n* = 16 Bivalirudin: *n* = 16	29 (5–128)	11 (4–56)	0.01
Machado et al. (29)	Heparin: *n* = 14 Bivalirudin: *n* = 18	12.54 ± 9.96	21.06 ± 12.53	0.06
Kaushik et al. (31)	Heparin: *n* = 27 Bivalrudin: *n* = 8	12 (5.75, 26)	14.5 (6.7, 16)	0.37

^a^Data displayed as mean ± standard deviation, median (interquartile range, i.e., 25th percentile, 75th percentile), or median (range, i.e., minimum–maximum).

### Transfusion requirements and bleeding events

Transfusion requirements were compared between pediatric patients receiving heparin or bivalirudin while on ECMO in four of the seven studies that were reviewed (Table 5). Transfusions of packed red blood cells (PRBCs), platelets, cryoprecipitate, and fresh frozen plasma (FFP) were evaluated in all four studies (28–30, 32). AT administered, as a categorical variable or quantified, was evaluated in two of seven studies (28, 29). Most studies found no significant differences in the quantity of PRBC, platelets, cryoprecipitate, or FFP transfused between groups though trends toward higher transfusion requirements were frequently noted in heparin cohorts. One of four studies in each of these blood product categories found a difference between groups. One of two studies reporting AT administration reported quantitative amounts of AT transfused, but only reported these values for the heparin group and did not provide statistical comparisons (29). The other study only reported AT administration as a categorical variable and found that more patients in the heparin group required AT (28).

**TABLE 5:** Transfusion requirements.

Study	Sample size	Heparin	Bivalirudin	*p*-Value
**PRBC**				
Seelhammer et al. (30)	Heparin: *n* = 65 Bivalirudin: *n* = 24	<24 h: 2 (1,3)^a^	<24 h: 2 (1,3)^a^	0.76
		24 h–7 days: 5 (2,15)^a^	24 h–7 days: 3 (1,11)^a^	0.15
Schill et al. (32)	Heparin: *n* = 34 Bivalirudin: *n* = 14	7.1 (3.2, 14.7)^b^	7.34 (5.3, 12.4)^b^	0.32*
Machado et al. (29)	Heparin: *n* = 14 Bivalirudin: *n* = 18	≤1 year of age: 1.51 ± 2.06^c^	≤1 year of age: 0.75 ± 0.41^c^	0.3734
		>1 year of age: 1.10 ± 1.28^c^	>1 year of age: 0.34 ± 0.18^c^	0.4750
Hamzah et al. (28)	Heparin: *n* = 16 Bivalirudin: *n* = 16	17.8 (3.4–75)^d^	7 (0–27)^d^	0.003
**Platelets**				
Seelhammer et al. (30)	Heparin: *n* = 65 Bivalirudin: *n* = 24	<24 h: 1 (1,2)^a^	<24 h: 1 (1,2)^a^	0.86
		24 h–7 days: 6 (2,13)^a^	24 h–7 days: 3 (1,6)^a^	0.04
Schill et al. (32)	Heparin: *n* = 34 Bivalirudin: *n* = 14	3.8 (0.0, 11.6)^b^	4.7 (1.4, 11.8)^b^	0.41*
Machado et al. (29)	Heparin: *n* = 14 Bivalirudin: *n* = 18	≤1 year of age: 1.07 ± 0.63^c^	≤1 year of age: 0.94 ± 0.54^c^	0.7645
		>1 year of age: 0.40 ± 0.34^c^	>1 year of age: 0.31 ± 0.38^c^	0.4743
Hamzah et al. (28)	Heparin: *n* = 16 Bivalirudin: *n* = 16	6 (1.6–34)^d^	5 (0–12)^d^	0.24
**Cryoprecipitate**				
Seelhammer et al. (30)	Heparin: *n* = 65 Bivalirudin: *n* = 24	<24 h: 1 (1,2)^a^	<24 h: 1 (1,2)^a^	0.89
		24 h–7 days: 2 (1,4)^a^	24 h–7 days: 1 (1,2)^a^	0.15
Schill et al. (32)	Heparin: *n* = 34 Bivalirudin: *n* = 14	0.0 (0.0, 0.3)^b^	0.5 (0.1, 1.1)^b^	0.047*^e^
Machado et al. (29)	Heparin: *n* = 14 Bivalirudin: *n* = 18	≤1 year of age: 0.09 ± 0.09^c^	≤1 year of age: 0.14 ± 0.07^c^	0.1868
		>1 year of age: 0^c^	>1 year of age: 0.0 ± 0.01^c^	0.7076
Hamzah et al. (28)	Heparin: *n* = 16 Bivalirudin: *n* = 16	0 (0–11)^d^	0 (0–5)^d^	0.38
**Fresh frozen plasma**				
Seelhammer et al. (30)	Heparin: *n* = 65 Bivalirudin: *n* = 24	<24 h: 1 (1,3)^a^	<24 h: 1 (1,3)^a^	0.47
		24 h–7 days: 2 (1,4)^a^	24 h–7 days: 3 (2,6)^a^	0.19
Schill et al. (32)	Heparin: *n* = 34 Bivalirudin: *n* = 14	0.4 (0.0, 9.6)^b^	1.2 (0.4, 4.1)^b^	0.86*
Machado et al. (29)	Heparin: *n* = 14 Bivalirudin: *n* = 18	≤1 year of age: 0.51 ± 0.64^c^	≤1 year of age: 0.20 ± 0.21^c^	0.0840
		>1 year of age: 0.20 ± 0.18^c^	>1 year of age: 0.06 ± 0.11^c^	0.3583
Hamzah et al. (28)	Heparin: *n* = 16 Bivalirudin: *n* = 16	8.5 (0–24)	0 (0–15)	0.001
**Antithrombin (quantified)**				
Machado et al. (29)	Heparin: *n* = 14 Bivalirudin: *n* = 18	≤1 year of age: 0.68 ± 1.16^f^	≤1 year of age: N/A	N/A
		>1 year of age: 0^f^	>1 year of age: N/A	N/A
**Antithrombin administered**				
Hamzah et al. (28)	Heparin: *n* = 16 Bivalirudin: *n* = 16	8 (50)^g^	2 (13)^g^	0.02

PRBC, packed red blood cell.

^a^Blood products (U), median (25th, 75th percentile).

^b^Blood products (ml/kg/day), median (IQR).

^c^Blood products (ml/kg/h), mean (SD).

^d^Blood products (ml/kg/day), median (minimum–maximum) or *n*(%).

^e^One heparin patient was transferred on ECLS to a burn center on the day of cannulation. This patient was excluded from survival analysis, both to decannulation and to discharge.

^f^AT units adjusted per hour of ECMO.

^g^*n*(%).

*Value may not be representative of direct comparison between groups.

The study reporting a higher percentage of the heparin group requiring AT while supported on ECMO was the analysis by Hamzah and colleagues (28), which found that 50% (*n* = 8) of the heparin group versus 13% (*n* = 2) of the bivalirudin group received AT (*p* = 0.02). This was also the study that found differences in the amount of PRBCs and FFPs administered. They found that patients anticoagulated with heparin required more PRBC (median [range], ml/kg/day: 17.8 [3.4–75] vs. 7 [0–27], *p* = 0.003) and more FFP (median [range], ml/kg/day: 8.5 [0–24] vs. 0 [0–15], *p* = 0.001). However, they reported no difference in the amount of platelets and cryoprecipitate administered between groups.

The study by Seelhammer and colleagues (30) compared 65 patients anticoagulated with heparin and 24 patients anticoagulated with bivalirudin at a single center and categorized transfusions provided to pediatric ECMO patients within the first 24 h of starting anticoagulation and transfusions given between 24 h and 7 days after the start of anticoagulation. When categorized as such, the authors found no differences in transfusion requirements within the first 24 h; however, between 24 h and 7 days, the heparin group required more platelets (median [IQR], units: 6 [2, 13] vs. 3 [1, 6], *p* = 0.04). There were no other differences between groups when categorized this way.

Schill et al. (32) did not directly compare transfusion requirements between those who were started on heparin or bivalirudin as the primary anticoagulant; they included a third group of patients who were switched from heparin to bivalirudin in their analysis of transfusions. Thus, *p*-values reported may not be representative of a direct comparison between those who were started on heparin or bivalirudin as their primary anticoagulant; however, non-significant *p*-values reported for the three group comparison allow us to conclude no statistical difference between these two groups, and significant p-values allow us to conclude that there may be a difference between these two groups though the difference could instead be in relation to the third group. The single-center study found that among 34 ECMO runs of patients anticoagulated with heparin and 14 ECMO runs of patients anticoagulated with bivalirudin (and 8 ECMO runs of patients switched from heparin to bivalirudin), there were no differences in PRBC, FFP, or platelet transfusions between the study cohorts (32). A statistically significant difference in cryoprecipitate was reported (*p* = 0.047), with the primary bivalirudin group receiving more cryoprecipitate than the heparin group (median [IQR], ml/kg/day: 0.5 [0.1, 1.1] vs. 0 [0, 0.3]); however, we cannot conclude whether this difference was between the heparin and bivalirudin groups or between either of these groups and the group that switched from heparin to bivalirudin.

Machado et al. (29) stratified transfusion requirements in the heparin and bivalirudin cohorts by age group (1 year or younger and older than 1 year) as transfusion thresholds were different in infants above and below 1 year of age. No differences were observed in each of the blood products used when stratified by age group.

When reviewing bleeding events, four of the seven studies evaluated bleeding events between pediatric patients receiving heparin or bivalirudin during ECMO (Table 6) (28, 29, 31, 32). Most of these studies found higher rates of bleeding among heparin cohorts though only one of these studies reached statistical significance. Hamzah and colleagues defined bleeding events as central nervous system bleeding or bleeding that required surgical intervention and found that the heparin cohort had a significantly higher rate of bleeding with 75% (*n* = 12) of patients experiencing at least one bleeding event compared to 18.8% (*n* = 3) in the bivalirudin cohort (*p* = 0.029) (28). The heparin group had 4 (25%) patients with no episodes, 4 (25%) patients with 1 episode, 4 (25%) patients with 2 episodes, 3 (18.8%) patients with 3 episodes, and 1 (6.2%) patient with 5 episodes. The bivalirudin group had 13 (81.2%) patients with no episodes, 2 (12.5%) patients with 1 episode, and 1 (6.3%) patient with 2 episodes.

**TABLE 6:** Hospital mortality and survival statistics.

Studyt7	Sample size	Heparin	Bivalirudin	*p*-Value
**Hospital mortality**				
Seelhammer et al. (30)	Heparin: *n* = 65 Bivalirudin: *n* = 24	37 (57)	10 (42)	0.20
Rabinowitz et al. (33)	Heparin: *n* = 35 Bivalirudin: *n* = 32	13 (37)	11 (34)	0.82
Schill et al. (32)	Heparin: *n* = 34 Bivalirudin: *n* = 14	12 (36)^a,b^	6 (46)^c^	0.91*
Machado et al. (29)	Heparin: *n* = 14 Bivalirudin: *n* = 18	6 (43)	6 (33)	0.5809
Hamzah et al. (28)	Heparin: *n* = 16 Bivalirudin: *n* = 16	5 (31)	3 (19)	0.62
Ranucci et al. (27)	Heparin: *n* = 5 Bivalirudin: *n* = 5	4 (80)	4 (80)	N/A
**Survival to decannulation**				
Schill et al. (32)	Heparin: *n* = 34 Bivalirudin: *n* = 14	24 (73)^a^	9 (64)	0.91*
Machado et al. (29)	Heparin: *n* = 14 Bivalirudin: *n* = 18	8 (57)	16 (89)	0.0396
Kaushik et al. (31)	Heparin: *n* = 27 Bivalirudin: *n* = 8	20 (74.1)	3 (37.5)	0.091
Hamzah et al. (28)	Heparin: *n* = 16 Bivalirudin: *n* = 16	11 (69)	13 (81)	0.62*
Ranucci et al. (27)	Heparin: *n* = 5 Bivalirudin: *n* = 5	2 (40)	3 (60)	N/A
**One-month survival**				
Machado et al. (29)	Heparin: *n* = 14 Bivalirudin: *n* = 18	8 (57)	13 (72)	0.3730
**Six-month survival**				
Machado et al. (29)	Heparin: *n* = 14 Bivalirudin: *n* = 18	8 (57)	12 (67)	0.5809

Values presented as *n* (%) unless otherwise specified.

^a^One heparin patient was transferred on ECLS to a burn center on the day of cannulation. This patient was excluded from survival analysis, both to decannulation and to discharge.

^b^One heparin patient was decannulated, re-cannulated on bivalirudin, and died.

^c^One patient remained hospitalized after two ECLS runs on bivalirudin.

*Value may not be representative of direct comparison between groups.

The other three studies reporting bleeding events had different definitions for bleeding events and reported higher, though not statistically significant, rates of bleeding in heparin cohorts. Machado et al. (29) defined major bleeding events as bleeding resulting in reoperation including mediastinal washout, or intervention on cannulation site or any type of surgical or specialized intervention, end-organ hemorrhage or dysfunction, or death resulting from bleeding. Five patients in the heparin group required nine mediastinal washouts compared to one patient in the bivalirudin group requiring two mediastinal washouts. One patient in the bivalirudin group also had epistaxis and arterial cannula site bleeding. This study did not provide a statistical comparison of these bleeding events between groups. The study by Schill and colleagues comparing three groups found no significant difference in the rate of hemorrhagic stroke between groups with a rate of 12% (*n* = 4) in the heparin group and 7% (*n* = 1) in the bivalirudin group. Lastly, Kaushik and colleagues defined major bleeding as the need for mediastinal/cannula site exploration for control of bleeding, bleeding requiring factor VIIa administration for control, gastrointestinal, pulmonary, or intracranial hemorrhage, or any bleeding requiring blood transfusion (31). Though not statistically significant, the bivalirudin group had a lower incidence of major bleeding at 12.5% (*n* = 1) compared to 44.4% (*n* = 12) in the heparin group (*p* = 0.21).

### Thrombotic complications

Out of seven studies, four measured the number of thrombotic complications between pediatric patients receiving heparin or bivalirudin while on ECMO (Table 7). Most studies trended toward a higher rate of thrombosis in the heparin cohorts. Out of the four studies, two did not find a significant difference in thrombotic complications between the two groups (28, 30), and two did not provide a statistical comparison (27, 29). One of the studies that did not report a statistical comparison had a small sample size with only five patients in each group; one patient in the bivalirudin group experienced a thrombotic complication (27). The other study reported a higher thrombotic complication rate of 29% (*n* = 4) among 14 patients anticoagulated with heparin compared to 5.6% (*n* = 1) among 18 patients anticoagulated with bivalirudin (29). Three of the four studies reported the specific complications that occurred (28–30), and an additional one study reported ischemic stroke rate but not overall thrombotic complication rate (32). There were no differences in the specific thrombotic complications that occurred between groups. The most common thrombotic complication reported was ischemic stroke, occurring in up to 21% of patients anticoagulated with heparin (32) and in up to 13% of patients anticoagulated with bivalirudin (30).

**TABLE 7:** Thrombotic complications on ECMO.

Study	Sample size	Heparin	Bivalirudin	*p*-Value
**Thrombotic complication, n (%)**				
Rannuci et al. (27)	Heparin: *n* = 5 Bivalirudin: *n* = 5	0 (0)	1 (20)	N/A
Hamzah et al. (28)	Heparin: *n* = 16 Bivalirudin: *n* = 16	3 (18.8)	0 (0)	0.1
Machado et al. (29)	Heparin: *n* = 14 Bivalirudin: *n* = 18	4 (29)	1 (5.6)	N/A
Seelhammer et al. (30)	Heparin: *n* = 65 Bivalirudin: *n* = 24	14 (22)	3 (13)	0.54
**Thrombotic complications, specified**				
Hamzah et al. (28)	Heparin: *n* = 16 Bivalirudin: *n* = 16	Ischemic stroke: 2 (12.5)	Ischemic stroke: 0 (0)	0.49
		Circuit thrombosis: 1 (6.25)	Circuit thrombosis: 0 (0)	
Schill et al. (32)	Heparin: *n* = 34 Bivalirudin: *n* = 14	Ischemic stroke: 7 (21)	Ischemic stroke: 1 (7)	0.67^a^
Machado et al. (29)	Heparin: *n* = 14 Bivalirudin: *n* = 18	LV clot: 2 (14)	LV clot: 0 (0)	N/A
		Ischemic limb: 2 (14)	Ischemic limb: 1 (5.6)	
Seelhammer et al. (30)	Heparin: *n* = 65 Bivalirudin: *n* = 24	Ischemic stroke: 11 (17)	Ischemic stroke: 3 (13)	0.75
		DVT: 6 (9)	DVT: 0 (0)	0.19

Values presented as *n* (%) unless otherwise specified.

^a^Schill et al. compares three groups: heparin, bivalirudin, and switched. The *p*-value represents a comparison between all three groups rather than between heparin versus bivalirudin.

### Mortality

Six of the seven studies evaluated hospital mortality, survival to decannulation, one-month survival, or six-month survival (Table 8) (27–30, 32, 33). Out of six studies comparing hospital mortality, none found a difference between the heparin and bivalirudin groups. Hospital mortality across all studies ranged from 31 (28) to 80% (27) in the heparin group and 19 (28) to 80% (27) in the bivalirudin group. The study that found hospital mortality to be 80% in both groups had a small sample size of five patients in each group and was the oldest study in this review, published in 2011 (27). The next highest reported hospital mortality rates were 57% (*n* = 37) (30) in the heparin group and 46% (*n* = 6) (32) in the bivalirudin group.

**TABLE 8:** Bleeding events.

Study	Sample size	Heparin	Bivalirudin	*p*-Value
**Bleeding events**				
Hamzah et al. (28)	Heparin: *n* = 16 Bivalirudin: *n* = 16	No episodes: 4 (25)	No episodes: 13 (81.2)	0.029
		1 episode: 4 (25)	1 episode: 2 (12.5)	
		2 episodes: 4 (25)	2 episodes: 1 (6.3)	
		3 episodes: 3 (18.8)	3 episodes: 0 (0)	
		5 episodes: 1 (6.2)	5 episodes: 0 (0)	
Machado et al. (29)	Heparin: *n* = 14 Bivalirudin: *n* = 18	Chest washout: 5 (36)*	Chest washout: 1 (5.6)**	N/A
		Epistaxis: 0 (0)	Epistaxis: 1 (7.1)***	
		Arterial cannula site: 0 (0)	Arterial cannula site: 1 (7.1)***	
Schill et al. (32)	Heparin: *n* = 34 Bivalirudin: *n* = 14	Hemorrhagic stroke: 4 (12)	Hemorrhagic stroke: 1 (7)	1^a^
Kaushik et al. (31)	Heparin: *n* = 27 Bivalirudin: *n* = 8	Major: 12 (44.4)	Major: 1 (12.5)	0.21
		Minor: 1 (3.7)	Minor: 1 (12.5)	0.41

Values presented as *n* (%) unless otherwise specified.

^a^Schill et al. compares three groups: heparin, bivalirudin, and switched. The *p*-value represents a comparison between all three groups rather than between heparin versus bivalirudin.

*Nine events in five patients.

**Two events in one patient.

***The same patient had epistaxis and arterial cannula site bleeding.

Five studies reported survival to ECMO decannulation—one study found significantly higher survival in bivalirudin patients (29), one study found lower though not statistically significant survival in bivalirudin patients (31), two studies found no difference in survival to decannulation (28, 32), and one study did not provide a statistical comparison (27). In the study reaching statistical significance, the bivalirudin cohort had 89% (*n* = 16) survival to decannulation compared to 57% (*n* = 8) in the heparin cohort (*p* = 0.0396) (29). This conflicts with the findings of Kaushik and colleagues’ (31) study in which survival to decannulation was 37.5% (*n* = 3) in the bivalirudin cohort versus 74.1% (*n* = 20) in the heparin cohort (*p* = 0.091). However, this study had a small sample size in the bivalirudin cohort of only eight patients compared to 27 patients in the heparin group. Omitting heparin or bivalirudin cohorts with less than 10 patients, survival to decannulation ranged from 57 (29) to 74.1% (31) in heparin cohorts and from 64 (32) to 89% (29) in bivalirudin cohorts.

Machado et al. (29) also compared 1- and 6-month survival between the heparin and bivalirudin groups and found no significant differences in either. Survival at 6 months was 57% (*n* = 8) in the heparin cohort and 67% (*n* = 12) in the bivalirudin cohort (*p* = 0.5809).

### Anticoagulation and ECMO costs

Out of the seven studies included in this review, one study evaluated total costs of anticoagulation, and another evaluated daily costs of ECMO (Table 9) (27, 28). Both studies found that pediatric patients supported with ECMO with bivalirudin used as the primary anticoagulant had lower healthcare costs. Hamzah et al. (28) evaluated total costs of anticoagulation while on ECMO, which included administration of anticoagulant and AT, as well as laboratory costs. They found the total cost of heparin administration was significantly higher than that of bivalirudin (median [minimum-maximum], dollars per day: 1,184 [83–7,142] vs. 493 [283–1,007], *p* = 0.03). Ranucci et al. (27) examined daily costs of ECMO care, which included the cost of the anticoagulant (bivalirudin vs. heparin), allogeneic blood product costs, and purified AT cost. The bivalirudin cohort also had significantly lower daily costs than the heparin cohort (mean ± SD, euros: 312 ± 56 vs. 760 ± 237, *p* = 0.008).

**TABLE 9:** Costs associated with ECMO and anticoagulation.

Study	Sample size	Heparin	Bivalirudin	*p*-Value
**Total costs of anticoagulation, $ per day** ^a^				
Hamzah et al. (28)	Heparin: *n* = 16 Bivalirudin: *n* = 16	1,184 (83–7,142)	493 (283–1,007)	0.03
**Daily costs of ECMO, € per day** ^a^				
Ranucci et al. (27)	Heparin: *n* = 5 Bivalirudin: *n* = 4	760 ± 237	312 ± 56	0.008

ECMO, extracorporeal membrane oxygenation.

^a^Data displayed as mean ± standard deviation, median (interquartile range, i.e., 25th percentile, 75th percentile), or median (range, i.e., minimum–maximum).

ECMO, extracorporeal membrane oxygenation.

## Discussion

Optimal anticoagulation therapy is necessary for the prevention of adverse outcomes in pediatric patients while on ECMO. While heparin has been the anticoagulant of choice in pediatric patients on ECMO, the use of bivalirudin as an alternative has recently become an attractive option. Although there are studies comparing heparin with bivalirudin in pediatric ECMO patients, there continues to be uncertainty regarding the optimal approach for reducing adverse thrombotic and bleeding events in this patient population. In our review of literature comparing heparin and bivalirudin use for ECMO therapy in the pediatric population, most patients included in these studies were young and small and were supported for postcardiotomy support, respiratory failure, or extracorporeal cardiopulmonary resuscitation. Results were largely heterogeneous among studies included. All studies reporting time to reach therapeutic anticoagulation reported conflicting results, aligning with the different anticoagulation targets between sites. Transfusion requirements, bleeding events, and thrombotic complications tended to be higher in pediatric ECMO patients anticoagulated with heparin though statistical significance was not consistently achieved. Mortality during and following ECMO support was mostly similar between heparin and bivalirudin cohorts. Lastly, anticoagulation with bivalirudin in these patients was associated with cheaper anticoagulation and ECMO costs.

Bleeding events are a frequent and unfortunate complication associated with ECMO support in pediatric patients. The Extracorporeal Life Support Organization (ELSO) reports that up to 31% of pediatric ECMO cases are complicated by surgical or cannula site bleeding and 11% of pediatric patients have an intracranial hemorrhage while on ECMO (34). Previous studies have evaluated bleeding complications associated with heparin and bivalirudin. In a cross-sectional survey of 22 Level IV NICUs involved in a neonatal ECMO focus group across the Children’s Hospital Neonatal Consortium from May to July 2022, DiGeronimo and colleagues reported that the 14 centers that incorporated bivalirudin into patient care were found to have fewer bleeding events, longer circuit life, and fewer transfusion of blood products, albeit one site experienced increased circuit complications with bivalirudin (35). Similarly, in a meta-analysis of nine retrospective studies with 994 adult and pediatric patients, of which five studies included pediatric patients, Mei et al. (36) found significantly lower risks of major bleeding in pediatric patients on bivalirudin versus heparin (RR: 0.27, 95% CI 0.11–0.0.64, *p* = 0.003). In our review, while only the study by Hamzah et al. (28) reached significance, most studies reported higher rates of bleeding in heparin patients, corroborating prior literature that bivalirudin may be a safer alternative associated with fewer bleeding events.

Inherent to ECMO circuits is the risk of thrombosis (37, 38). In 2019, ELSO reported that thrombotic complications occurred in up to 16.7% of neonatal and 12.4% of pediatric patients (39). Existing literature suggests that thrombotic complications may also be lower in pediatric ECMO patients on bivalirudin compared with heparin. Liu et al. (40) conducted a systematic review and meta-analysis of 14 retrospective studies with 1,501 patients. Their study included mostly adult patients with only five studies reporting outcomes in pediatric patients. In a subgroup analysis of the 210 pediatric patients included, the authors found significantly reduced risk of thrombosis in pediatric ECMO patients on bivalirudin compared with heparin (OR = 0.38, 95% CI 0.16–0.92, *p* = 0.031). Though statistical significance was not achieved in the individual studies included in our review of 313 pediatric patients, likely partly due to the lack of statistical analysis in some studies, our study, which includes a larger sample, largely supports the findings of Liu and colleagues that bivalirudin may be associated with a lower rate of thrombotic complications among pediatric ECMO patients.

Mortality in pediatric ECMO patients represents a major clinical challenge due to complications such as bleeding and thrombosis associated with ECMO therapy (39). These complications can account for up to 30–40% of fatalities in pediatric ECMO cases (34). Although most studies in our review did not find a difference in hospital mortality between heparin and bivalirudin groups, existing literature favors fewer events of in-hospital mortality in the aggregate of pediatric and adult patients anticoagulated with bivalirudin compared with heparin. In a systematic review and meta-analysis of ten retrospective studies with a total of 847 adult and pediatric patients, Ma et al. (12) found lower in-hospital mortality in ECMO patients on bivalirudin versus heparin (*I*^2^ = 0%, *p* = 0.007, OR = 0.64, 95% CI: 0.46–0.88). However, their analysis of in-hospital mortality combined results of pediatric and adult patients and included only four pediatric studies with a total of 177 pediatric patients. Thus, this analysis is mostly reflective of in-hospital mortality in adult patients. In contrast to the study by Ma and colleagues, our review summarizes a larger number of pediatric ECMO studies and provides a more granular analysis of mortality in pediatric patients, including survival to decannulation, in-hospital mortality, 1-month survival, and 6-month survival. In our review, granular analysis of these endpoints suggests no difference in hospital mortality, 1-month survival, or 6-month survival, and conflicting results for survival to decannulation.

While our study suggests that bivalirudin may be associated with lower rates of bleeding and thrombosis with no difference in mortality when compared to heparin in pediatric ECMO patients, bivalirudin is a more expensive anticoagulant than the readily accessible heparin (41). As such, hospitals and patients may not feel inclined to incorporate bivalirudin into treatment plans. Surprisingly, both studies reporting a cost analysis in our review (27, 28), found significantly lower total costs of anticoagulation and daily costs of ECMO, respectively, in pediatric patients treated with bivalirudin compared to heparin (27, 28). Current literature discusses the potential barriers that bivalirudin poses to ECMO patients due to its high price. In a retrospective cohort study with 15 pediatric ECMO patients on bivalirudin, Campbell and colleagues found bivalirudin medication to be five times the cost of heparin. Importantly, the authors note that cost of medication is only one component of patients’ healthcare bills, which also includes “the number of circuit changes, additional use of AT and blood products, and laboratory testing” (42). Our review suggests that bivalirudin may be a more cost-effective option than heparin when considering the overall costs of anticoagulation. Perhaps this is due to the observed decreases in bleeding and thrombotic complications and/or transfusion requirements when patients are treated with bivalirudin. Alternatively, this may be attributed to the cost of AT replacement, which contributes significantly to the cost of using heparin. Variability in AT replacement across institutions would be expected to impact the difference in cost of using these two agents; however, both studies evaluating cost in our review highlight the cost-effectiveness of bivalirudin. Yet, cost alone should not determine the anticoagulant used and should only be interpreted within the context of overall outcomes achieved with each anticoagulant.

Our study has limitations. All studies included in our systematic review were retrospective in nature, thus introducing the possibility of selection bias. Patients anticoagulated with bivalirudin may have had a history of intolerance to heparin or have received bivalirudin as the primary anticoagulant of choice. A senior ECMO team member (Peek) cared for patients in both Kaushik and Machado’s studies. Although most studies included in our review had comparable baseline characteristics between groups, children placed on ECMO have many comorbidities and a wide range of diagnoses, making comparisons challenging. In addition, there are institutional differences between study sites regarding cannulation strategies and cannula sizes used, and we were unable to control for this variability. Institutional differences in indications for bivalirudin versus heparin also limit the generalizability of our findings. By including only comparator studies, we aimed to limit the effect of these limitations.

## Conclusion

In our review of pertinent literature comparing heparin and bivalirudin use for ECMO therapy in the pediatric population, we found that bivalirudin-based anticoagulation is safe, efficacious, and cost-effective. While heparin is indicated in many settings and may be acceptable for certain pediatric patients requiring ECMO support, bivalirudin may offer decreased risk of bleeding and thrombotic complications and lower transfusion requirements with a lower overall cost in this setting. Anticoagulation targets differ between institutions, and mortality during and following ECMO support appear to be similar. Prospective multicenter studies with standard anticoagulation targets are needed to more accurately compare the characteristics and outcomes associated with bivalirudin versus heparin in pediatric ECMO patients.

## Data availability statement

The original contributions presented in this study are included in the article/supplementary material, further inquiries can be directed to the corresponding authors.

## Author contributions

GP, EJ, OS, and CV contributed to the conception and design of the study. CV and OS organized the database. CV, OS, MM, and RS wrote the first draft of the manuscript. All authors contributed to manuscript revision, read, and approved the submitted version.
